# An exploration of clinical practice in sites with and without clinical nurse or midwife specialists or advanced nurse practitioners, in Ireland

**DOI:** 10.1186/s12913-016-1412-8

**Published:** 2016-04-26

**Authors:** Imelda Coyne, Catherine M. Comiskey, Joan G. Lalor, Agnes Higgins, Naomi Elliott, Cecily Begley

**Affiliations:** School of Nursing and Midwifery, Trinity College Dublin, 24 D’Olier Street, Dublin 2, Ireland

**Keywords:** Clinical specialist, Advanced practitioner, Nurses, Midwives, Case study, Clinical practice

## Abstract

**Background:**

Clinical specialist (CS) and advanced practitioner (AP) roles have increased in nursing and midwifery internationally. This study explored clinical practice in sites with and without clinical nurse or midwife specialists or advanced nurse practitioners in Ireland.

**Methods:**

Using a case study design, interview, observational and documentary data from postholding sites (CSs or APs employed) were compared with data from non-postholding sites (no CSs or APs employed). Interviews and observations were conducted with postholders (*n* = 23), and compared with data from healthcare professionals (nurses or midwives, doctors) (*n* = 23) in matched services. Interviews were held with Directors of Nursing and Midwifery (*n* = 23), healthcare professionals (*n* = 41), service users (*n* = 41) with experience of receiving care or working with postholders, and non-postholders in matched services. The data were analysed using Nvivo (Version 8).

**Results:**

The findings suggest that postholders’ practice appeared to differ from non-postholders’ in relation to case management and service provision. Postholders were seen as having an impact on readmission rates, waiting lists/times, collaborative decision-making, continuity of care and workload management. Postholders’ autonomy to manage caseloads was perceived to lead to smoother transition of patients/clients through the healthcare system. Service-users’ self-reports appeared to appreciate the individualised holistic care provided by postholders. Postholders’ role in facilitating person-centred care and promoting interprofessional team working, are essential elements in quality care provision and in global healthcare workforce planning.

**Conclusions:**

To meet changing healthcare demands, promote person-centred care, and improve service delivery, more specialist and advanced practice posts in nursing and midwifery should be developed and supported within healthcare.

## Introduction

Over the past four decades, specialist and advanced practice roles in nursing and midwifery have expanded rapidly internationally [1, 2]. The roles have been in existence in Canada [3, 4] and the United States [5] for over 40 years but have only been introduced in Europe more recently [6]. Since the introduction of these roles, there appear to have been substantial increases in the type and numbers of advanced practice roles globally due to healthcare restructuring, burgeoning healthcare needs, healthcare workforce supply and demand and government policy and support [7–9].

Likewise, in Ireland, nursing has undergone significant change over the past decade in relation to the clinical role and responsibilities of nurses and midwives [10]. Clinical nurse or midwife specialist roles developed in response to health service restructuring, identified service needs, a national reduction in junior doctors’ working hours, potential for nurses and midwives to enhance patient care and outcomes, and the expansion of nurse and midwife-led clinics [11–14]. Robust frameworks and accreditation processes for advanced practice posts, with mandatory site preparation and development of posts to meet population and service needs, were established in Ireland [15–17]. The term ‘postholder’ refers to a nurse who is a clinical nurse/midwife specialist or an advanced nurse/midwife practitioner in a specific service.

## Background

In Ireland, the organisation responsible for creating and supporting APs and CSs posts, called the National Council for the Professional Development of Nursing and Midwifery (NCNM), established a framework that clearly outlined the roles and requirements for ANP/AMPs and CNS/CMSs [15, 17]. Since 2010, the Irish Nursing and Midwifery Board of Ireland (NMBI) has been given the statutory responsibility for the regulation and accreditation of APs and CSs. In the framework, the core competencies of advanced nurse practitioners (ANP) are: autonomy in clinical practice, expert practice, professional and clinical leadership and research [15, 18]. These attributes are consistent with the attributes of APs internationally [19]. They are distinguished from clinical specialists by their advanced clinical nursing or midwifery knowledge, level of autonomous decision-making, responsibility, and accountability in caseload management [15]. Advanced practitioners are required to have a Master’s degree or higher in nursing or midwifery and have at least 7 years post qualification experience, including 5 years in their specialist area of advanced practice [15, 16].

Clinical nurse and midwife specialists (CNS/CMS) are defined by specialist clinical focus, patient/client advocacy, education and training, audit and research and consultancy [17]. A key part of their role is the ability to assess, plan, initiate and evaluate treatment according to agreed protocols [17]. The CSs do not have the same level of autonomy in caseload management compared to APs. Clinical specialists are required to have a Higher Diploma (post-registration qualification) in nursing or midwifery and have at least 5 years post qualification experience, which should include 2 years in their clinical area of specialist practice. APs and CSs can prescribe medicinal products and ionising radiation provided they have completed an approved course in both topics [20–22]. The clinical practice role is arguably the most important and has been shown to take up the majority of Clinical Specialist (CS) 1/Advanced Practitioner 2(AP)’s time [23–25].

Specialist and advanced practice roles are seen as enhancing service delivery and building the capacity of the nursing and midwifery professions, but the impact of these roles is often invisible [26, 27]. Evaluating the effectiveness of advanced practice roles is challenging [28], as it is difficult to measure impact since the role often has both direct and in-direct impact on health outcomes. However, within the last decade there has been a significant increase in international evidence that demonstrates the contribution of CS/APs roles on patient care in hospital, primary and long-term settings [24, 29–34]. In America, Newhouse et al. [34] systematically reviewed the evidence to determine the impact on patient outcomes of advanced practice registered nurses (APRN) compared to other providers (doctors or teams without an APRN). The APRN term included nurse practitioners (NP), clinical nurse specialist (CNS) and certified nurse midwives (CNM). The review included 69 studies and concluded that patient outcomes of care provided by NPs and CNMs in collaboration with doctors were similar to, and better in some cases than, care provided by doctors alone. They also found that, within acute care settings, CNSs could reduce length of stay and cost of care.

Van Soeren et al. (2011) used mixed methods (observation, self-recorded logs of consultations, focus group interviews with team members and 46 NPs) to investigate the NP role across a range of clinical settings in Canada [24]. They concluded that NPs were key to the delivery of patient centred care (in terms of accessibility and patient safety) and interprofessional practices. Although some small scale evaluation of the AP and CNS/CMS role in Ireland has occurred [35, 36], the roles had not been evaluated nationally. Therefore a study was commissioned to evaluate the role and outcomes of the Clinical Nurse and Midwife Specialists (CNS/CMS) and Advanced Nurse and Midwife Practitioners (ANP/AMP) in Ireland [37]. The findings on the differences between CS and APs’ roles [38], leadership roles and factors influencing leadership roles [39, 40], the views of policy-makers, predictors for service users’ outcomes [41], concept analysis of ANP [19], using case study design [42], research and audit outcomes for CS [43] and service user outcomes [44] have been published in a series of papers. One paper illustrated how case study design is a very appropriate methodology to use when evaluating the impact of complex roles and interventions in health care outcomes and service delivery [42].

With regard to leadership, the findings indicated that the CS/AP’s ability to perform a leadership role was mediated by four key factors, including the personal attributes of postholders, professional development, opportunities to act as a leader, and support to sustain leadership [39]. Ten key activities were identified for leadership at advanced practice level, seven regarding clinical leadership and three for professional leadership [40].

With regard to differences between APs and CSs, the key findings were: that APs provided improved service delivery, and showed greater leadership, increased research and a clear governance and accreditation structure, compared with CSs. Clinical Midwife Specialists were rated more highly for ‘continuity of care and carer’ than both CNSs and APs [38].

The findings indicated that policy-makers in Ireland believe that CS/AP roles contributed to higher quality care through a more holistic approach, improved continuity of care, better patient/client outcomes, more new initiatives in care, and greater staff education, audit and policy/guideline development [41]. Furthermore that CS/APs provided more evidence-based practice and conducted audit more frequently than did other clinicians working in comparable clinical roles in matched sites [43]. The logistic modelling of the key outcome of the SCAPE study, which was “noticing any difference in the care given by CS/APs compared with care given by other clinicians” showed that the single most important predictor was described as “being treated with respect” [44]. The findings from the CS/APs’ clinical practice role are reported in this paper and suggest that CS/APs practice appeared to differ from non-postholders in relation to case management and service provision.

## Aim and objectives

This paper reports on a small section of the larger study (Specialist Clinical and Advanced Practitioner Evaluation in Ireland or “SCAPE” study), which was a focused evaluation of the clinical services provided by clinical nurse and midwife specialists and advanced nurse and midwife practitioners in Ireland. The research objectives was to explore how the clinical practice aspects of the postholder CS/APs might differ from those of non-postholder health providers and the key objectives were:

1. To explore the provision of care received in sites with CS and AP and without from the perspective of service users, clinicians and decision makers.
2. To explore clinicians’ perceptions of care given to service users in sites with and without accredited clinical specialist and advanced practitioner post-holders.

## Methods

To investigate the activities of CSs and APs and the context in which they practised, a case study approach [45] was seen as most suitable as it is flexible method that emphasises multiple sources of data, recognises multiple realities and captures meaningful characteristics of real life events [45, 46]. The case study approach was deemed suitable for investigating a contemporary topic (CSs and APs roles, skills, impact) in a real life situation (healthcare settings) where it could be difficult to extract practices from the effects of the social context [42]. In doing case study research, the case being explored can be an individual, organisation or event, which may be, time and place specific. In this study the phenomenon of interest was specialist and advanced practice. The case was the organisation or site where nurses or midwives worked, namely, the ward, community or clinic. The cases were matched based on the clinical speciality i.e. a diabetes service with CNS in diabetes was compared with a diabetes service without a CNS in post.

### Sample and sites

A total of 23 CS/APs were compared with 23 non-postholders (nurses or midwives, or doctors) providing a service in similar services in matched non-postholding areas. ‘Postholding sites’ were sites where CS or AP worked and those without such posts were called ‘non-postholding sites’ or ‘matched sites’. All postholders had to be approved by the NCNM and have at least 1 year in post. The postholders (17 CSs and six APs) were purposively chosen from 13 sites, providing representation nationally and from all the disciplines (Table 1).

**Table 1:** Postholders and non-postholders sample

Discipline	Advanced Practitioners (APs) Clinical Specialists (CSs)	Non-postholders and matched sites
Midwifery	0 APs (None in post)	3 Clinicians, hospital & clinics: prenatal screening service, diabetes care team, infectious diseases team
	3 CSs, hospital & clinics (Diabetes care, prenatal screening, infectious diseases)	
General nursing	3 APs, hospital sites (Emergency department (ED), sexual health, endoscopy)	9 Clinicians, hospital sites: stroke care services, pulmonary outreach service, heart failure clinic, anticoagulation therapy service, pain management care team, colposcopy, ED, sexual health clinic, endoscopy clinic
	6 CSs, hospital sites (Pulmonary outreach, stroke care, heart failure, anti-coagulation therapy, pain management, colposcopy)	
Mental health nursing	1 AP, community site (Child & adolescent mental health)	5 Clinicians, community sites: child and adolescent mental health team, family therapy team, cognitive behavior therapy service, psychotic disorders team, addiction service.
	4 CSs, community sites (Addictions, cognitive behaviour therapy, psychotic disorders, family therapy)	
Children’s nursing	1 ANP, hospital site (Emergency department)	1 Clinician, hospital site: Emergency department
	1 CNS, hospital site (Ear, nose and throat clinic)	1 Clinician, clinic: ear, nose, and throat clinic
Public health	1 AP community site (Practice nursing)	1 Practice nurse: community service
	1 CS community site (Care of the elderly)	1 Clinician: care of the elderly community service
Intellectual disability nursing	0 APs (None in post)	2 Clinicians, residential units: challenging behaviour team, early intervention services.
	2 CSs, residential units (challenging behaviour, early intervention)	
Total	6 APs and 17 CSs = 23 Postholders	23 clinicians in matched sites

Data were collected through non-participant observations and interviews with post-holders (CS/APs), non-postholders (12 Clinical Nurse Manager (CNM) Grade 2 and 3,^3^ 2 nurses, 2 midwives, 1 behaviour therapist and 8 Consultant/registrar/senior house officers), healthcare professionals (Directors of Nursing or Midwifery (DON/DOM), nurses, doctors) and service users (patients/clients, family members/carers) who had direct contact with CS/APs or with the non-postholders (see Table 2).

**Table 2:** Data Collection: Methods, Participants, and Tools

Data collection method	Postholding sites (*n* = 13)	Matched sites (*n* = 15)	Total participants
Observation of the postholder and non postholder for two 2-h sessions over 1 week, as they delivered care and interacted with service users and other staff.	23 postholders	23 healthcare professionals providing services (12 Clinical Nurse Manager Grade 2/staff-nurses, 2 midwives, 1 behaviour therapist and 8 doctors)	46
Interviews were held with nurses and doctors from six areas: midwifery (*n* = 4), and children’s (*n* = 3), general (*n* = 18), community (*n* = 4), mental health (*n* = 8) and intellectual disability nursing (*n* = 4).	21	20	41
Interviews were held with service users from five areas: midwifery (*n* = 6), and children’s (*n* = 4), general (*n* = 18), community (*n* = 4), and mental health nursing (*n* = 9).	20	21	41
Interviews (Director of Nursing or Midwifery) from six different areas: midwifery (*n* = 3), and children’s (*n* = 2), general (*n* = 7), community (*n* = 2), intellectual disability (*n* = 3) and mental health nursing (*n* = 6).	8 (plus 5 postholding and matched)	10 (plus 5 postholding and matched)	28

### Ethics

Approval was obtained from the Faculty Research Ethics Committee Trinity College Dublin and the local Ethics Committee in all sites. Following ethical approval, information packs were posted to identified post-holders, and their matched counter-parts in all non postholding sites, outlining the purpose of the study, nature of participation and enclosing an invitation to take part. Once consent was received, a research assistant contacted the respondent directly to arrange a suitable time to observe practice and interview participants. Informed consent was obtained from all participants, including patient/clients and other healthcare professionals involved when a CS/AP or non-post-holder was giving care while being observed. Confidentiality was ensured through using codes for names and sites, removal of identifying information, and strict data storage.

### Data collection

#### Interviews

Semi-structured interviews were conducted with service users/family members/carers (*n* = 41), healthcare professionals (*n* = 41) and directors of nursing or midwifery (*n* = 23) from post-holding and non-postholding sites. Interviews were conducted in a quiet location, digitally recorded and ranged from 10 to 25 min. The interview schedules contained open questions focused on areas of care that were phrased to elicit both positive and negative experiences (see Table 3). For example: what was good about your care and what was not so good about your care? Questions were phrased in simple words when interviewing children, or those with intellectual disability, to facilitate understanding. All participants were given the opportunity to add further information at the conclusion of the interview.

**Table 3:** Interview topics and questions

Participant groups	Topics	Examples of questions
Interviews with clinicians working with APs/CSs, or in the non postholding teams (10 to 45 min)	Co-workers’ understanding of CS/AP role Teamwork and communication Best practice in service delivery Care pathways Research awareness	What do you understand the Clinical Nurse/Midwife Specialist (or Advanced Nurse Practitioner) role to be? How you have experienced inter-disciplinary team-work and communication between yourself, the CS/AP and the rest of the team? To what extent has the CS/AP role influenced the adoption and/or implementation of evidence-based care, implemented national health policy or clinical guidelines? In relation to care pathways, questions were asked about the appropriateness of aspects of care that included: assessment and diagnosis, interventions, referral/liaison, initiating/ending health care episodes. How would you describe the level of knowledge about research in your unit, team or ward?
Interviews with Directors of Nursing and Directors of Midwifery (27 to 45 min)	CS/AP role Teamwork and communication Best practice in service delivery Staff education and support Research awareness	Have you noticed any difference in the service provided since the CNS/ANP was/were appointed? How do you view inter-disciplinary team-work and communication between the CS/AP and the rest of the team? Has the CS/AP role influenced the adoption and/or implementation of evidence-based care, implemented national health policy or clinical guidelines in their area? Has the CS/AP contributed to the education of other staff (nurses/midwives/others) in this hospital/service? How would you describe the level of knowledge about research in the CNS/CMS/ANP’s area?
Interviews with service users (10 to 25 min)	Communication Relationships Continuity of care & Access to care Satisfaction with care Any difference in care given by postholders	Please tell me how you have experienced communication between yourself and your clinician – what were the good things, and the not-so-good things ?. And the relationship between you and your clinician? How was that? Please tell me how easy or difficult you feel your access to care was? Any examples you can give me? Was there good continuity of care (seeing same person/few people each time?) In general, how satisfied are you with the care given by your clinician? How does she/he help you to manage your …..(disease, condition, symptoms)? Have you noticed any difference in the care given by the CS/AP compared to care given by other members of the health care team?

The observation tools and interview schedules were piloted, which resulted in minor amendments to the observation schedule. To ensure validity, the four research assistants underwent training in how to use the tools to identify activities that represented specialist or advanced practice.

#### Non-participant observations

Using a structured observation schedule, the postholders (*n* = 23) and non-postholders (*n* = 23) were observed for two 2-h sessions over 1 week as they delivered care and interacted with service users and other staff to identify evidence of actions occurring under three categories (see Table 4). The 4 h of observation of each postholder and non-postholder resulted in a total of 184 h. The four researchers also recorded actions such as communication skills, safety aspects, use of research evidence and education of patients/clients in a “key behaviours” score sheet (Table 5). This was developed from the literature on items of good practice, and tested with the four researchers, using video scenarios, until complete accuracy was achieved. The score sheet was then used in a pilot study of two sites, without any changes being necessary. The context in which care was delivered, namely the identification of relevant structures available to support the post-holder, the care pathways followed by the service user and any relevant local policies were recorded in field notes.

**Table 4:** Observation schedule

Type of outcome	Outcome	Example of evidence/lack of evidence
Patient	Patient/client involved in shared decision making	
	Patient/client/family knowledgeable and prepared (preparedness for treatment, patient or family education, health promotion activity	
	Patient/client aware of diagnosis and understands consequences, treatment options,	
	Facilitates continuity of care	
Nurse or midwife or health professional	Advises others on use of evidence/research relevant to practice	
	Involved in policy development/dissemination	
	Involved in knowledge-educating other professionals	
	Provides leadership in an area of practice	
	Involved in clinical initiatives (care pathway development, clinical guidelines)	
	Addresses patient/client symptoms and experiences of illness/distress addressed (Symptom management, looking at holistic assessment, assessment that is wide ranging)	
	Demonstrates clinical autonomy	
Health Care Service	Evidence of shared decision making with other members of the multi-disciplinary team	
	Refers to other health professionals	
	Assessment of service needs initiated by the CNS or ANP	
	Facilitates speedy access to services for patient/client	
	Evidence of networking/linking with community health professionals/voluntary organisations on patient/client issues

**Table 5:** “Key behaviours” score sheet

Communication					
Criterion	A	F	S	N	Notes
* Listening skills* – clinician gives time for patient/client to talk, looks open and relaxed, shows by response that they have heard what was said					
*Feedback* – clinician checks that patient/client understood what was said					
*Decision making* – patient/client’s point of view asked for, patient/client appears involved in decision					
*Information giving* – gives information either verbal, written, or by demonstration					
*Using open questions* – clinician picks up and acts on cues “You look distressed…”, or “Is there anything you would like to ask?”					
*Liaison with other key stakeholders* (family, other MDT, other and state which)					
Safe environment					
Criterion	A	F	S	N	Notes
*Hand washing* – between every patient/client					
*Using gloves*, if appropriate					
*Equipment* – maintaining sterility					
Using research evidence					
Criterion	A	F	S	N	Notes
*Refers to research*, or evidence from audit, or web-sites, during consultations					
Health promotion/lifestyle					
Criterion	A	F	S	N	Notes
*Health promotion* advice or literature given – in addition to information on the specific disorder/reason for care					
*Education provided* on self-monitoring the patient/client’s condition					

A = Always, F = Frequently, S = Sometimes, N = Never, Notes = Notes on evidence – how condition was met. N/A can be used for “Not applicable”

#### Analysis of documentary evidence

A quantitative summary of documentary evidence (audits, postholders’ work diaries, work-programmes, etc.) on each site was also included. The work diaries and programmes were borrowed by the four RAs and photocopied, with permission. They showed clinic times and dates, numbers of patients/clients seen, meetings attended, committees contributed to, conferences attended, teaching given, consultations provided and any other activities. The evidence was constantly referred back to during the analysis and write-up phases to seek corroboration of salient points brought out in the interviews or observational data.

### Data analysis

The data from the observations, interview transcripts, and documentary evidence were imported into, managed and analysed using the Computer Assisted Data Analysis Software programme (CAQDAS) NVivo V8© [47]. Four independent external experts in advanced practice research tested (by taking a sample of data and analysing it using the framework) and consequently confirmed the validity of the framework used. Then the researchers (three lecturers in nursing and midwifery) worked together in coding the activities undertaken by the postholder and non postholder in clinical practice, which were applicable to both CSs and APs. The data formed two themes, which were underpinned by five categories.

## Results

The data on clinical practice formed two themes that were: case management, and service provision. The findings are presented under these two main themes and categories and supported with participants’ quotes and observational data (Table 6).

**Table 6:** Themes and categories

Themes	Categories	Concepts
Case management	Assessment & diagnosis Referral	Managing the care pathway Multidisciplinary team-work Record-keeping Administration
Service provision	Developing therapeutic communication	Service user satisfaction with a good relationship.
	Health promotion: education of service user & family	Improved health knowledge of service users and carers.
	Physical & psychosocial interventions	Health outcomes.

### Theme 1: case management

The postholders appeared to practise differently from non-postholders in the areas of assessment and diagnosis, and referral. These two categories included the concepts of managing the care pathway, multidisciplinary teamwork, record keeping and administration.

#### Assessment and diagnosis

The assessment and diagnosis of clients’ needs was viewed as a key part of all postholders’ role. Assessment was seen as thorough because of postholders’ in-depth knowledge of, and good relationships with, the patients/clients and the utilisation of available resources. Thorough assessments were seen by doctors as enabling successful treatment or appropriate referral, and contributing to a reduction in hospitalisation and unnecessary tests for clients.

*The nurse would know the patient quite well. On a more person-to-person level. They would have an idea regarding the patient, sometimes some patients could be worrying for nothing and if you know them for a while on a personal basis, it helps a lot. It doesn’t mean that they would be ignored but at least in terms of prioritising should they be seen quickly in the clinic or not (Interview, Doctor, postholder site, CNS).*

When clients required referral, APs were noted for their skill in ensuring quick action by communicating with relevant professionals and/or by ordering relevant tests and investigations.

*We had a case 2 weeks ago of a chap in his seventies who was referred for investigation of anaemia. At diagnosis he had a tumour. So [AP] immediately got the surgeon to have a look at it and then contacted the nurse specialist to arrange all the outpatient CT scans…exactly what you would expect someone to do in a responsible position, automatically it was all done (Interview, Doctor, postholder site, AP).*

The coordination and liaising between professionals within the multi-disciplinary team (MDT) was seen by senior nurses and doctors as promoting good patient/client care and wellbeing.

*A lot of the respites would be support for carers, but an awful lot of our respites are clinically, there’s a clinical base to it as well and she (ANP) coordinates all that. She does all the pre-admission, assistance for respite and continuing care, all of the continuing care needs with the placement system and the fair deal system now…then into respite, she would liaise with the staff and, you know, say she needs this, she needs that, she needs the other (Interview, Director of Nursing, postholder site, ANP).*

The observational data revealed that postholders frequently assessed clients’ clinical and associated educational needs. Where the AP was a Registered Nurse Prescriber, observations and documentary evidence included prescribing relevant medication. For example, the summary of observations below illustrate the range of care provided by APs for clinical practice, and demonstrate holistic assessment.

ANP used several assessment tools – Wong Baker face scale, pain ladder, FLACC Scale (pain), Glasgow Coma Scale, Lund and Browder Chart, Modified Parkland Formula. Scope of practice guidelines re ANP minor injuries (paediatrics) developed by her in conjunction with consultants and benchmarked against international and national guidelines. Saw 5 clients per each 2-hour observation seen. – Each client’s pain levels checked at beginning of consultation and analgesia given promptly as required (Fieldnote observation, postholder site, ANP).

#### Referral

The postholders’ referral role featured frequently in the observation and interview data in relation to taking referrals directly and referral to other professionals such as the consultant, MDT members, community services, other hospitals, and general practitioners.

*They (CNS) will liaise with other members of the team. If they feel that a client needs to be looked at from another area, they would propose that… They work very closely with the psychiatrist, myself as well, but they would have all the assessments done, all the interventions done (Interview, CNM3, postholder site, CNS).*

In addition, the observational data revealed frequent referrals:

The CNS…refers to PHN and community GPs. She also refers to the medical team, audiologists and physiotherapists. (Fieldnote observation, postholder site, CNS).

The ANP has the autonomy to refer clients to other health care professionals. During the observation periods the ANP referred one client to the counsellor, one to the health advisor, one client to the consultant in Infectious Diseases. She also liaised with the laboratory personnel requesting various tests on samples she had taken (Fieldnote observation, postholder site, ANP).

Taking referrals directly appeared to be a key aspect of the AP role rather than the CS’s role and it was one that had to be negotiated and approved with other members of the multidisciplinary team and referral agencies.

*The ANP takes referrals, when they triage and do assessments, they actually make the decision and say, “This case requires a [health professional]. This case requires a CNS. This case requires childcare. This case requires a [specialty doctor].” So, they as a nurse are making that decision. They don’t make it solely alone. Obviously for best practice they have to consult and it wouldn’t be wise not to, but they do make the decisions and I don’t know of any cases really where they were second guessed or told, “No, that’s not right,” It’s worked extremely well (Interview, Director of Nursing, postholder site, ANP).*

#### Non-postholding sites

With regard to non-postholder sites, it was evident that assessment was also taking place; but there was less emphasis on knowing the service user as a person, and more healthcare professionals appeared to be involved in the assessment process, which delayed diagnosis and commencement of treatment. The caseload was also different, with variance in both types of activities performed and numbers of clients seen. In addition, non CS/AP nurses conducted a series of tasks for multiple patients, rather than giving holistic and complete care to a smaller number.

*We don’t have a CNS in [health problem]. In that case it would be dealt with by the most appropriate member of the team. Most likely…the consultant and the community [specialty] nurse. If the person required hospitalisation it would be dealt with by…the medical team with nurses on the ward and they would be treated and discharged to respective community services as soon as possible (Interview, Director of Nursing, non postholder site, matched CNS).*

Within the non-postholder sites, the consultants mainly initiated the referrals, and there was mention of some inappropriate referrals, and of the need for nurse to “suggest” referrals rather than being able to refer patients themselves.

*When the patient is admitted again at our report we’ll decide they need physio, they need OT…we will suggest these referrals should be done. We’ll get the team and say ‘this patient needs physio’ or ‘needs OT’ or whatever. So we ensure those referrals are made to the appropriate team (Interview, CNM3, non postholder site, matched CNS).*

### Perceived differences between postholders and non-postholders in relation to case management

The postholders were perceived to make a difference with regard to: readmission rates, collaborative decision-making, continuity of care, waiting lists/waiting times and workload management.

#### Readmission rates

Postholders (CS and APs) were perceived to reduce readmission rates by providing advice about symptoms, linking with General Practitioners (GPs) in their area, and identifying when clients needed admission before they deteriorated.

*It (an audit of practice) indicated here they (CNS) are involved in the management of a client, they might have an earlier admission before the symptoms deteriorate. Their stay is shorter and they would have less admissions (Interview, Assistant Director of Nursing, postholder site, CNS).*

These perceptions were supported by documented fieldnotes from the observational data.

*There was no such service prior to the CS taking on the role. Prior to the CS, [symptom] was a common reason for a person [with cardiac problem] being an emergency admission to CCU…These admissions have now dramatically reduced (Fieldnote observation, postholder site, CS).*

Within non-postholder sites, participants did not mention any impact on readmission rates. One site mentioned the problems with having no dedicated CNS in a particular unit.

*This was originally set up as care of the elderly…it was meant to be for assessing patients as well, that they could come in on a booked basis and be assessed here, but again with…the shortage of beds…we don’t actually have any beds for patients who come in as booked admission for assessment. It now means the patients that are a bit sicker come to ED and then transferred. Whereas a lot of that could be avoided if the patient could come in and be seen while they’re good really (Interview, CNM3, non postholder site, matched ANP).*

#### Collaborative decision-making

Although postholders appeared to have significant autonomy in referral and treatment, there was evidence of multidisciplinary (MDT) collaborative decision-making.

*She’s (CS) excellent. She provides a lot of nurse-led services and she’s making decisions and advising on the care of our patients. Knowing they always have full support from myself and that any questions she’d contact myself and I’d be available to help her with her decision-making (Interview, Doctor, postholder site, CS).*

Within the non-postholder sites, there was evidence of communication and good team working in all the disciplines. However, team working appeared to be consultant-led rather than nurse- or midwife-led and there appeared to be more references to following guidelines rather than undertaking autonomous, client-centred care, as was found in CS/AP sites.

*For example, blood sugars…those are done in the first 48 h and if they are within the normal range, they can be discontinued. The same then with neuro obs…for the first 48 h they’re carried out and if everything is okay they can be discontinued, but we have it all in the care pathway, so regardless of who is going to mind that patient it’s all there to follow (Interview, CNM 3, non postholder site, matched ANP).*

In addition, the “key behaviours” score sheet showed that 96 % of postholders (*n* = 22) always involved clients in decision-making, compared with 67 % (*n* = 14) of observed clinicians in the non-postholding sites, while 96 % (*n* = 22) liaised with key stakeholders (Directors of Nursing or Midwifery, and members of the clinical team) compared with 71 % (*n* = 15) of non-postholders.

#### Continuity of care

Continuity of care appeared to be enhanced because CS/APs were there permanently whilst the junior doctors and registrars who were fulfilling the same role were usually in an area only for a specific length of time. Postholders were perceived to acquire knowledge of clients/patients over longer periods of time and to provide continuity of care, which was highly valued by service users.

*She (AP) sees the patient and does the full screen. Then whatever problem they have she will do the health advice as well and the [prevention] whereas they see a doctor and he’ll do the screen and have the next patient in. They might refer them on to a health advisor or social worker. AP is all those people in one. Doctor is just a doctor. No disrespect (Interview, Nurse, postholder site, AP).*

Service users in postholder sites frequently commented on how they appreciated the continuity of care and how they valued the fact that their ‘story’ was known and did not have to be repeated constantly. Some doctors noted that the continuity of care provided by postholders made their role easier.

*It’s very helpful because you know her and she knows your history and all that. Yes, we have a very good relationship. (Interview, Service user, postholder site, AP).*

These views were also documented in the observation fieldnotes. Knowing the postholders appeared to contribute to service users’ increased attendance at clinic appointments, and to more holistic care.

The CNS has experienced 2–3 % DNA (Did Not Attend) rates compared to considerably higher in the traditional model where clients tend to DNA more (up to 30 %) where there are a number of different doctor clinicians on each visit…[the CNS is] able to maintain more continuity of care (Fieldnote observation, postholder site, CNS).

Within the non-postholding sites, there were comments from healthcare professionals also about how the lack of a dedicated CNS created difficulties with maintaining continuity of care. Some service users in non-postholder sites commented on how they found it difficult to keep repeating information to different nurses, and how they found the lack of continuity stressful.

#### Waiting lists and waiting times

The postholders’ screening role was seen as helping to reduce waiting times and ensure quicker throughput of patients/clients. It was suggested that the postholders’ autonomy enabled them to progress clients through their care more swiftly, which ensures that large clinics can be managed efficiently.

*If we didn’t have the accredited nurse [procedure], there’s no way we would be anywhere near what we are. If you look at the statistics of how many [procedures] are done by nurses compared to doctors, it’s startling. Most of the work, the majority, is done by the nurse (Interview, CNM3, postholder site, CS).*

This opinion was substantiated by the audits conducted in the adult ED site, which provided written evidence of reduction in waiting times since ANPs were appointed. In colposcopy services, there was documentary evidence of waiting lists being reduced in postholding sites from previous levels. Postholders manage clients alongside doctors, which, in the view of participants, ensures that large clinics can be progressed quickly.

In non-postholding sites, there were some comments made in relation to the length of waiting times, and of trying to reduce waiting lists and experiencing frustration but no audits were available in these sites to support these comments.

#### Workload management

Doctors and other members of the MDT in different specialties spoke about postholders contributing significantly to the workload management.

*They have an essential role to play and we need more nurse specialists. There’s such a shortage of them. If you could just get more support, more nurses getting trained in these areas, and funding for them. It is just so badly needed (Interview, Doctor, postholder site, CS).*

These views on the amount of work undertaken by postholders were substantiated by fieldnotes.

Thirty three clients seen by CNS (total number seen at full 3 h clinic = 68 − staff nurse in attendance for 90 min and admin assistant for 60 min), 2 bleeps, 2 phone calls, 1 query in person by family member, liaison with CNS [similar health issue] × 3 occasions (Fieldnote observation, postholder site, CNS).

Postholders were observed to manage their workload independently and the caseload appointment system. The autonomy and decision-making ability of the postholders was seen as contributing to clients being cared for swiftly and efficiently, thus increasing the daily throughput.

*For me it would mean much more responsibility and pressure because having [AP] here is like a huge buffer in that she’s so independent, she’s so expert, she’s so reliable. She’s technically excellent. She has that extra knack that you can’t teach…when she’s here you know that things are going to run right and everything is running as it should. When she’s not here, you feel the pressure coming on…everything comes to you (Interview, Doctor, postholder site, AP).*

In the non-postholding sites, there were a number of comments in relation to lacking support to manage the workload efficiently and to having sufficient time for patients/service users.

*I feel that because of the pressure of the list being so busy and the pressure of time and of the fact that we don’t have enough staff, we don’t actually get involved with the patients. They are beginning to be a number (Interview, CNM2, non-postholder site, matched CS).*

### Theme 2: service provision

The postholders were identified as differing from the non-postholders in relation to therapeutic communication; health promotion: education of service user and family; and physical and psychosocial interventions.

#### Developing therapeutic communication

The data indicated that postholders develop good relationships with patients/clients, and their carers, because they gave people time, listened to concerns and showed empathy.

*The nurse can carry out the procedure the same way a consultant would do, but it’s the empathy they would have with the patient and it’s the whole communication thing (Interview, Director of Nursing, postholder site, ANP).*

These data were substantiated by fieldnotes and also noted by other clinicians working with postholders.

The CNS facilitates the nurse-led clinic for review of [health issue]. During observation of 45 min the CNS reviewed 3 patients. Patient no 1: The CNS took a detailed history, examined [body part] and obtained a swab for microscopic culture and sensitivity. The carer was informed regarding the infection and was involved in the decision-making processes. The patient was reviewed by the doctor at the CNS’s request and commenced on oral antibiotics with the full understanding of the carer (Fieldnote observation, postholder site, CNS).

These skills were noted by other clinicians working with postholders and, in some comments, by service users who expressed appreciation of their expertise.

*Well, I’m quite a quiet person. I wouldn’t have very much to say. She’s very, very good at talking; explaining things, going through things, asking me questions (Interview, Service user, postholder site, CS).*

Within the non-postholding sites, there were no comments in the interviews in relation to developing therapeutic communication. This is not to say it does not happen but perhaps there was less emphasis on the notion of communication as ‘therapeutic’. Communication skills noted on the “key behaviours” scoresheet showed that 96 % of postholders (*n* = 22) always used good listening skills, gave feedback and used open questions compared with 67 % (*n* = 14), 81 % (*n* = 17) and 81 % (*n* = 17) of observed clinicians in the non-postholding sites.

#### Health promotion: education of service user and family

Postholders were perceived to be very good at providing information, support and education of service users. It was reported that service users could contact postholders on a range of matters such as: advice on new symptoms, issues of concern, and to clarify doctor’s communication.

*She does an awful lot of patient education and carer education, so whether she’s with a patient in the clinic or she’s seeing her inpatients, she will also see a relative or somebody (Interview, CNM3, postholder site, CS).*

The documentary evidence showed that information leaflets were available in postholder and non postholder sites. However additional resources (*n* = 63) had been developed in 16 sites by postholders, specifically for their services compared to non-postholding sites. For example:

The ANP gave relevant leaflets on how to care at home, when and who to contact as appropriate. She advised one [patient] about need for an operation the next morning for nail bed injury, demonstrating autonomous practice. She gave full advice on fasting, where and when to come to hospital and what to expect. She checked they understood and gave the opportunity to discuss any concerns (Fieldnote observation, postholder site, ANP).

Postholders’ health promotional activities included the provision of educational materials and practical teaching activities that focused on increasing the service users’ knowledge.

*We looked at my x-rays together … it’s a hairline fracture on my thumb and she picked up on that. It could have been missed very easily and she did show it to me and explain it to me and she did follow through with practical advice [on what to do] if you have a fractured thumb, dietary intake and rest treatment, elevating the limb (Interview, Service user, postholder site, AP).*

Examples were provided of postholders organising and running patient/client education programmes on disease management, encouraging self-care and setting up self-help groups. This educative and health promotion role was repeatedly confirmed in the observational data and documentary evidence.

*When patients feel that they have had a good experience, in that they have more of a focus on holistic care than many of the junior doctors, maybe, so aspects of care including risk reduction and advice in terms of self care are much more expertly delivered by a group that’s focused in that area. (interview, Consultant doctor, postholder site, ANP).*

Within the non-postholding sites, there was more emphasis on information sharing rather than specific education and health promotion strategies.

*[Talking about improvements required] I suppose more time for the patient before they come. To have an assessment clinic so when they come they know what’s ahead of them, that they are not faced with something totally unknown.…she admits them and discharges them, so you have that continuity of care. Give them information when they are being discharged as well…Sometimes you just do a procedure and that’s it (Interview, CNM2, non postholder site, matched ANP).*

Results of the ‘key behaviours’ score sheet showed that higher percentages of postholders appeared ‘always’ to give information (91 %, *n* = 21 versus 81 %, *n* = 17), health promotion advice (65 %, *n* = 15 versus 19 %, *n* = 4) and education (61 %, *n* = 14 versus 24 %, *n* = 5). In addition, 26 % of postholders (*n* = 6) always referred to research when explaining care to patients/clients and 70 % (*n* = 16) frequently did, compared with 14 % (*n* = 3) and 70 % (*n* = 16) of non-postholders respectively.

#### Physical and psychosocial interventions

Participants noted how postholders used physical interventions to improve care for service users, such as: symptom management, physical comfort, pain relief, medication and nurse-led clinics.

The CNS was called to perform the first [procedure] on day 6 on a [patient], it is policy that the CNS undertakes the first [procedure] post operatively as she possesses the specialist knowledge and skills in this area of care at a higher level than a staff nurse (Fieldnote observation, postholder site, CNS).

Positive physical intervention initiatives included the establishment of advanced or specialist nurse or midwife-led clinic. Postholders also appeared to use psychosocial interventions to good effect in their care of patients and clients.

*I think she’s a very switched-on person and she’s very good at knowing when to say nothing. I really like her very much and I completely trust her. I think she’s excellent … I have seen at different times in my life different kinds of therapists, I think she’s probably the best person I’ve seen (Interview, Service user, postholder site, CS).*

These clients’ views were supported by fieldnotes taken during the observation periods, many of which showed evidence of holistic care.

The ANP demonstrated clinical decision-making – for example – a client’s finger tip injury needed plastics operation; the ANP decided not refer them for surgery today, but to come back in the morning fasting and better prepared for the operation – physically, psychologically and practically. (Fieldnote observation, postholder site, ANP).

Within the non-postholding sites, although physical and psychosocial interventions did occur, there were fewer descriptions of holistic care.

*While the girls [nurses] are working very well, they don’t have the time to be the expert and their main focus isn’t the [specialty] clinic. They have a wider remit for [task] in the hospital as a whole, so we have deficits in patient education, in research, in policy and pathway information…forging links with primary care. (Interview, CNM3, non-postholder site, matched CS).*

The “key behaviours” scoresheet showed differences in activities involved in the provision of physical interventions. These included adequate hand washing ‘always’ occurring between every patient/client contact for 61 % (*n* = 14) in postholding and 38 % (*n* = 9) in non-postholding sites, ‘always’ using gloves, 39 % (*n* = 9) versus 24 % (*n* = 5) and ‘always’ using equipment correctly, 53 % (*n* = 12) versus 43 % (*n* = 9).

### Perceived differences between postholders and non-postholders in relation to service provision

In the view of the majority of those interviewed (healthcare professionals, service users), and from the analysis of observations, fieldnotes and audits, postholders were perceived to differ from non postholders in the following areas of care: service user satisfaction with a good relationship, improved health knowledge of service users and carers, and improved health care.

#### Service user satisfaction with a good relationship

Service users reported that they valued a good relationship with postholders, which led to feelings of trust in their capabilities and trust that they would be seen as persons. It appeared to be the personal touch and feeling that postholders were accessible that many appreciated, and other healthcare professionals commented on this also:

*We can ring her at any time. She is our first point of contact for anything. We ring her before we even ring the GP. We find that she knows us and what is best for us to do. When we left the hospital she encouraged us to follow up on the phone if we had any issues. She gives the best advice as she knows us the best so we rely completely on her (Interview, Service user, postholder site, CNS).*

Good relationships were viewed as contributing to satisfaction with care received.

*The atmosphere during the clinic was friendly with all patients greeting the CS fondly and most by name. She also knew them by name and their histories. She knew by name many of the family members also. Patients seemed to know the clinic routine and process for results well (Fieldnote observation, postholder site, CS*).

Within the non-postholding sites, it was evident from service users that they viewed the healthcare professionals as kind; however, there appeared to be less emphasis on having a therapeutic relationship with one key professional.

#### Improved health knowledge of service users and carers

Postholders were seen as contributing towards improving the health knowledge of service users. Service users appreciated postholders who could explain the procedures and investigations in clear, understandable language.

*A client attended the clinic and was seen by AP. A full and detailed discussion regarding treatment options and [specialty] health promotion was identified. Client asked a number of questions which were answered fully by the AP. Treatment was preformed and full post treatment advice was given verbally and in written form. Medications were given to the client and possible side effects, treatment regime and usage were given. Client commented that she felt confident in the advice she had been given and would follow through on it (Fieldnote observation, postholder site, AP).*

In non-postholding sites, there were numerous comments in relation to the need to employ postholders to improve service users’ knowledge and awareness.

*It’s not to say that the patients aren’t being treated well…they are, but if we had a CS in the hospital…part of that person’s role would be developing policies…sometimes people are discharged from the ward with a book… nothing written in it and turn up to [specialty] clinic…with no education whatsoever (Interview, CNM3, non postholder site, matched CS).*

#### Improved health care

Many participants reported that the postholders promoted good care for service users in the following ways: enhanced compliance with treatments, reduced readmissions, prompt treatment and reduction in problems worsening.

*When she took over the care of the [patients] you could see an immediate change in the control of [health problem], and it changed the philosophy of what the patients did for themselves. They were taking ownership of the care of their [health problem] and they were much more pro-active and understanding what they needed to do and not coming in a distressed illness state (Interview, Doctor, postholder site, AP).*

The postholders were perceived to deliver interventions through prompt and personalised care for service users.

*They (CNS) play a very important role… and make life very easy for the [cardiac problem] clinician…these patients would have the clinical nurses’ phone numbers and they could literally phone them at any time during the weekdays and give them advice over the phone or if there was any concern they could be seen, even before their due appointment…it actually prevents these patients deteriorating… and they are caught early and dealt with and treated quite promptly. They get medical attention at the right time (Interview, Doctor, postholder site, CNS).*

Service users in receipt of health promotion advice reported improved health, reduced attendance at healthcare centres and greater confidence to self-care:

*We ring her any time we need to and we also have regular appointments. Even if we are at other clinics…we call in to say hello. We look out for her when we go for our appointments and hope it is she that is there as she knows us best and we know her best so we are more comfortable with her. She gives us confidence to manage which is very important to us. I was initially attending 2 to 3 times a week but now I have 6 month appointments and that will show you how I have improved…because I do what I am advised, I take the advice seriously and it has made things so much better (Interview, Service user, postholder site, CNS).*

The postholders’ education and health promotion role was viewed as contributing to the maintenance of quality standards of care and serving as a role model for nursing and medical staff.

In the non-postholding sites, there appeared to be less emphasis on specific health outcomes; nevertheless, service users appeared satisfied with level of service.

*Absolutely fabulous. I couldn’t fault them. Even, just even the way of chatting to you before the birth of the twins. I found that not enough could be done for me. I had everybody coming into me, every consultant and you know, through to the counsellor popping in every single day (Interview, Service user, non postholder site, matched CNS).*

In non-postholding sites, there were more comments from healthcare professionals in relation to lack of time.

*They have spoken to me so they know me then when they come and that’s very good. But, you know, I don’t get the chance to do it constantly all the time. Most of the time I’m doing it but there’s the odd week I just don’t get time to do it because…when you get them on the phone it can take half an hour (Interview, CNM2, non postholder site, matched ANP).*

## Discussion

The study findings indicate that postholders (CS and APs) were perceived to differ from non-postholders in relation to case management and service provision. Postholders engaged in collaborative decision-making as characterized by their significant autonomy in referral and treatment, and reports of multidisciplinary (MDT) collaborative decision-making. Postholders promoted continuity of care as characterized by their permanent dedicated posts and acquired knowledge of clients/patients over longer periods of time. Some audits indicated that caseload management appeared to reduce readmission rates and waiting lists/times. These findings are similar to previous evaluation studies of these roles within Ireland (NCNM 2004, 2005) and previous studies from United Kingdom (UK) and United States of America (USA) [31, 48]. Although postholders were quite autonomous in referral and treatment, they nevertheless appeared to work closely with members of the multidisciplinary team (MDT) in making collaborative decisions, which has been reported elsewhere [24]. Members of the MDT appeared to value working collaboratively with postholders, and sharing perspectives on clinical care. Their comments indicated respect for postholders’ expertise and contribution to case management.

The findings suggest that medical staff did not see postholders as encroaching on their ‘territory’ as might have occurred in the past [49, 50]. Instead they appeared to value and respect the contribution of postholders to patient/client care, which has been reported in another Irish study of APs in Emergency departments [51]. This recognition of postholders’ contribution is to be welcomed as studies indicate that APs and doctors provide equally effective patient care and achieve similar health outcomes [34, 52, 53]. Numerous examples were provided of where postholders’ caseload management contributed towards the smoother transition of patients/clients through the healthcare system. Members of the MDT perceived postholders as ensuring continuity and cohesion because they act as the interface between all professionals involved in patient care. So postholders were seen as the ‘glue’ that coordinated care delivery and team working thus ensuring a ‘one stop shop’ for patient care. Similarly a study the UK found that ANPs were the ‘lynch-pin’, whose pivotal role facilitated both nursing and medical practice [54]. Likewise, a Canadian study found that NPs (see ANPs) provided a central coordinating role in the delivery of patient care [24]. In Hong Kong, a case study of advanced nurse practice led-clinics found that APs played a central role in the promotion of integrated teamwork within the MDT [55].

In relation to service provision, postholders were seen as differing from non-postholders in the area of therapeutic communication, health promotion, education of service user and family, the use of physical and psychosocial interventions and increased patient/client satisfaction, which has been reported elsewhere [28, 29, 34, 54, 56] and in previous evaluations of these roles [35, 36]. The focus on the patient/client as an individual and the provision of holistic timely care appeared to be key aspects of postholders’ contribution to service provision, which has been reported elsewhere [55, 57]. The findings indicated that postholders were seen as developing good relationships with patients/clients because they gave people time, listened to concerns and showed empathy. The importance of being treated with respect was reflected in a survey of service users from the same study [37], reported in an earlier paper [44]. From the patients/clients’ perspective, having sufficient time was appreciated because issues could be discussed more fully and acknowledged [58, 59].

Postholders were perceived as offering more holistic care because they could manage patient care from assessment to diagnosis and from treatment to discharge. Postholders were identified as differing from non-postholders in relation to service user satisfaction with a good relationship, improved health knowledge of service users and carers, and some health care. The findings provides clear descriptions of the important contribution that AP and CS make to care and how they function at an advanced level. Overall, the contributions to patient care achieved by the postholders in Ireland are comparable to the sub-roles of advanced practice identified in a systematic review of relevant studies (*n* = 42 studies) from Australia, UK and USA, such as expert holistic clinical care, direct interventions, care coordination, and being a patient advocate [60].

### Limitations

The findings must be treated with caution, as this was a qualitative exploration of roles and lacked hard data such as service audits to corroborate all the reported differences observed in practice, or gleaned from interviews. There were difficulties with matching some of the postholding sites in the field of intellectual disability with comparable clinical sites that cared for similar patients/clients, where no postholders (CS and APs) were employed. It is a limitation that where unique posts existed these could not be evaluated as the focus was on using matched comparisons. It is a limitation that the observational data were captured during certain time frames only, and consequently only represent a snapshot of what was occurring, and it is known that observers can alter usual behaviour. This is a limitation of the observation method, as the findings can only give information about those times that clinicians were observed and do not necessarily represent usual practice.

## Conclusions

Despite the limitations, this study appears to show a difference between the postholders and non-postholders particularly for case management and service provision, which is consistent with the international literature [34, 61]. The postholders appeared to played a key role in promoting interprofessional team working, which is essential in strengthening healthcare workforce globally [62]. The International Council of Nurses (ICN) sees CSs and APs as being central to delivering the WHO goal of ‘health for all’ and thus is committed to helping support more advanced nursing roles [2]. An international survey of the role in 32 countries concluded that NP/APNs ‘represent a sleeping giant for healthcare systems worldwide…to meet the need for increased access to quality health care’ ([1], p37). Likewise in the USA, advanced practice registered nurses (includes APs, CSs) play a critical role in healthcare reform and restructuring of a more effective healthcare system [9, 34]. Irish health policy and senior policy-makers value and endorse the strategic role of CS and APs in the delivery of a high quality patient-centred service in a variety of settings [41, 63]. Advanced practice roles should continue to develop in response to changing healthcare needs rather than as replacements for doctors working shorter hours, which have occurred in the past [64, 65]. To continue to meet the demands of high quality accessible healthcare in an era of cost containment, it is essential that CS and APs roles are supported and allowed to expand so that nursing and midwifery workforces are responsive to changing healthcare needs, demographic change, advances in care and treatment, new knowledge and technology, and increased expectations from service users and families.

### Ethics approval and consent to participate

Approval was obtained from the University Faculty Research Ethics Committee Trinity College Dublin and the local Ethics Committee in all sites.

### Consent for publication

Not applicable.

### Availability of data and materials

The dataset supporting the conclusions of this article contains clinical data, and informed consent for publication of the dataset was not gained from participants at the start of this project. As we have an ethical and legal responsibility to respect participants’ rights to privacy, and to protect their identity, the dataset cannot be published.

## Abbreviations

AMP: advanced midwife practitioner
ANP: advanced nurse practitioner
AP: advanced practitioner which includes both advanced nurse practitioner and advanced midwife practitioner
APN: advanced practice nurse
CMS: clinical midwife specialist
CNC: clinical nurse consultant
CNS: clinical nurse specialist
CS: clinical specialist which includes both clinical nurse specialists and clinical midwife specialists.
ENP: Emergency nurse practitioner
NCNM: National Council for the Professional Development of Nursing and Midwifery
NP: nurse practitioner

## Footnotes

1 Clinical Specialist (CS) will be used to encompass both clinical nurse specialists and clinical midwife specialists.

2 Advanced Practitioner (AP) will be used to encompass both advanced nurse practitioner and advanced midwife practitioner.

3 Clinical Nurse Manager Grade 2 and 3 refers to nurses who have at least 5 years post registration experience of which 2 must be in the specialty or related area.
